# Early-life weight gain patterns of term small-for-gestational-age infants and the predictive ability for later childhood overweight/obesity: A prospective cohort study

**DOI:** 10.3389/fendo.2022.1030216

**Published:** 2022-11-22

**Authors:** Ping Li, You Lu, Di Qie, Ling Feng, Guoqian He, Sufei Yang, Fan Yang

**Affiliations:** ^1^ Department of Pediatrics, West China Second University Hospital, Sichuan University, Chengdu, China; ^2^ Key Laboratory of Birth Defects and Related Diseases of Women and Children (Sichuan University) Ministry of Education, Chengdu, China

**Keywords:** infant, chidhood, small-for-gestational-age, obesity, weight gain

## Abstract

**Objectives:**

We aimed to identify the weight gain patterns of small-for-gestational age (SGA) infants in early life and to explore the predictive value for later overweight/obesity in childhood.

**Methods:**

We obtained data from a prospective cohort including term SGA infants born between January 2006 and November 2015 who received regular health care from birth to 5 years in West China Second University Hospital, Chengdu, China. A latent class growth analysis (LCGA) was applied to group children with similar growth trajectory patterns. Multiple logistic regression was performed to examine the association between weight gain patterns and later overweight/obesity.

**Results:**

A total of 296 term SGA infants were finally included. Five weight gain trajectories were identified, including excessive rapid catch-up growth (ERCG) (class 1, 10.9%), rapid catch-up growth (RCG) (class 2, 17.9%), appropriate catch-up growth (ACG) (class 3, 53.0%), slow catch-up growth (SCG) (class 4, 13.4%) and almost no catch growth (NCG) (class 5, 4.8%). SGA infants in class 1 and class 2 had a higher BMI according to age- and sex-specific Z scores from 2–5 years of age. In addition, 25% of SGA infants in class 1 and 13.2% of SGA infants in class 2 were found to be overweight/obese at 2-5 years of age. After adjusting for confounders, we found that extremely rapid weight gain (class 1) in the first 2 years of life increased the risk of overweight/obesity by 2.1 times at 2 to 5 years of age (aOR=2.1, 95% CI: 1.3~4.8; *P*<0.05). Furthermore, the increment of ΔWAZ between 0 and 4 mo was prominently related to the risk of overweight/obesity at 2 to 5 years for term SGA infants (aOR=3.2, 95% CI: 1.7~8.1; *P*<0.001). A receiver operating characteristic (ROC) curve showed the area under curve (AUC) was 0.7, with a 95% confidence interval (CI) from 0.6 to 0.8 (*P*<0.001).

**Conclusions:**

The extremely rapid weight gain pattern of term SGA infants in the first 2 years of life increased the risk of overweight/obesity at 2 to 5 years of age. It suggests monitoring weight gain across the infant period represents a first step towards primary prevention of childhood obesity.

## Introduction

Small-for-gestational-age (SGA) refers to newborns with birth weight (BW) below the 10th percentile according to the sex- and gestational age (GA)-specific reference (INTER- GROWTH-21st Project) (1). Globally, approximately 16% of all infants are SGA, and this figure ranges from 7% in industrialized countries to 41.5% in South Asia (2, 3). It was reported that 32.4 million infants (27% of live births) were born SGA in low- and middle-income countries in 2010, and among them, 29.7 million were term SGA infants (3). In recent study, it was reported that a third of babies were born SGA (34%) and SGA accounts for a quarter (24%) of all neonatal deaths in South Asia (4). The number of SGAs was huge, and it had a significant impact on children’s short-term and long-term health. Most term SGA infants showed significantly rapid weight gain or catch-up growth (CUG) compensating for intrauterine restraint within the first two years of life (5–7). However, associations between SGA and increased risks for disease in adulthood, such as metabolic syndrome, type 2 diabetes and cardiovascular disease, are now well established (8–11). Furthermore, growing evidence has suggested the greatest long-term risk of excessive adiposity and the accompanying comorbidities across life among infants who have been found to have intrauterine growth restriction followed by rapid weight gain in infancy (10, 12–15). Notably, increasing evidence has shown that children who experience rapid weight gain in early postnatal life are more prone to developing obesity and related diseases than those born SGA only (16).

Currently, obesity and accompanying comorbidities have become a global public health concern and have spread to the pediatric population (17, 18). In total, 35.1% of American children aged 2~19 years developed overweight/obesity from 2015~2016, and a sharp increase in obesity prevalence among children aged 2~5 years was detected (19). Treatment of overweight and obesity is notoriously difficult and often unsuccessful. Therefore, prevention-based strategies implemented as early as possible seem to have profound significance. As illustrated by the Developmental Origins of Health and Disease (DOHaD), obesity and accompanying comorbidities might originate very early from maternal, perinatal and early childhood factors; for example, SGA infants with postnatal accelerated weight gain were demonstrated to constitute an enormous high-risk group for developing overweight/obesity (20–22). Most studies have proposed that the most influential window for achieving catch-up growth is the first two years of life (5, 6, 23, 24). Thus, this window might be a critical period for setting the long-term growth trajectory and for the early prevention and intervention for overweight/obesity in later life.

To date, limited studies are available regarding the weight gain trajectories for SGA infants, especially in low-middle income areas, and a consensus has not been established regarding which period of weight gain contributes to future risks for overweight/obesity. Hence, we performed a prospective cohort study enriched with term SGA births to 1) identify weight growth patterns within the first 2 years of life for term SGA children; 2) explore the association of particular weight gain patterns with the development of overweight/obesity in later life; and 3) evaluate the predictive impact of differential rapid weight gain periods during infancy on later overweight/obesity.

## Methods

### Study design and subjects

**Figure 1:**
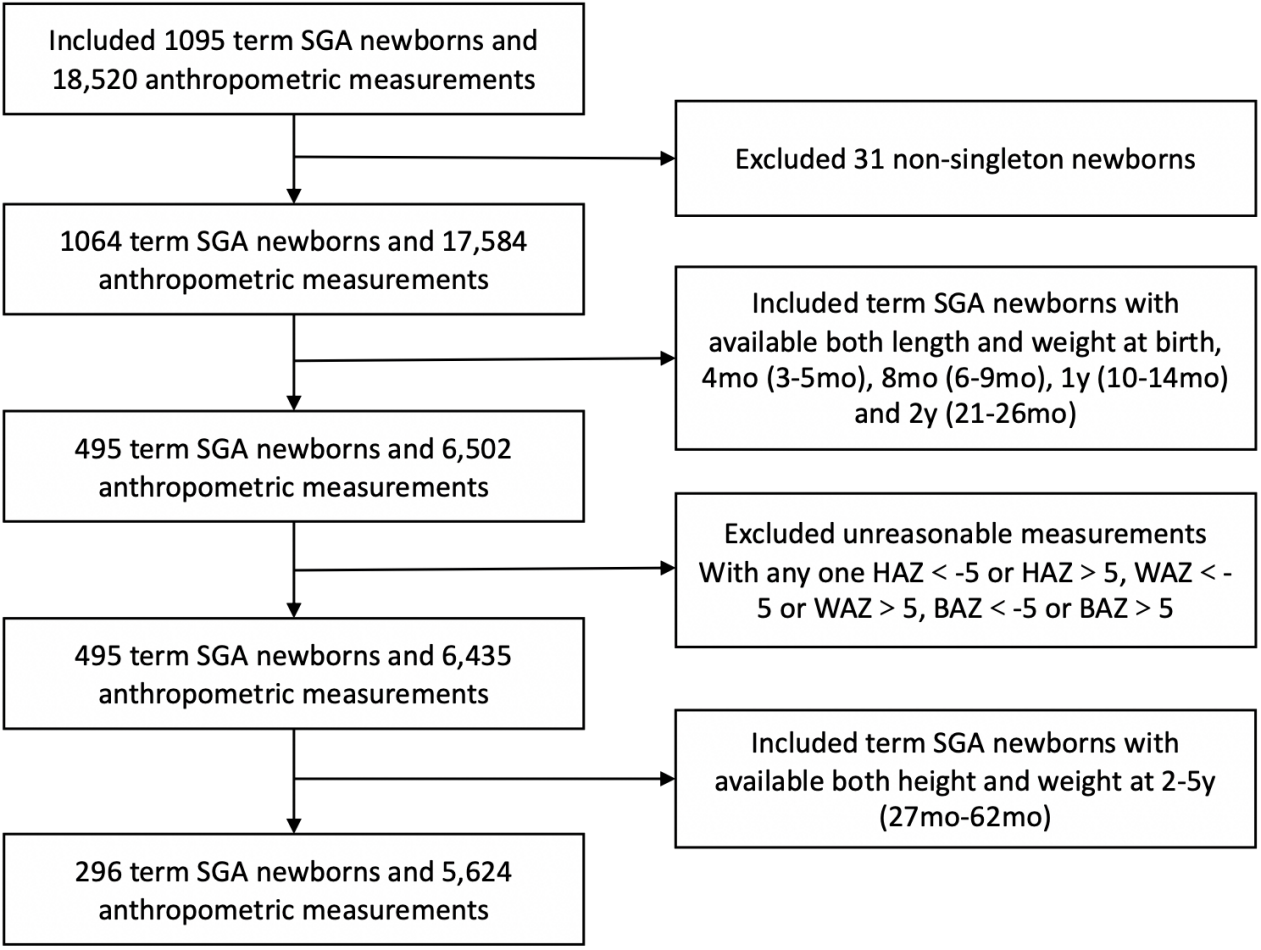
Flowchart of the study population.

Data were obtained from a cohort who had regular health care and birth data in the Children’s Department of Health at West China Second University Hospital, Chengdu, China. Children were born between January 2006 and November 2015. Gestational age (GA), birth weight (BW), sex, gravidity and parity were collected at birth. Feeding patterns between 0~4 mo were documented. Parental weight, height and calculated body mass index, as well as the parental education were recorded. In the study, SGA was defined as birthweight < 10th percentile for sex and gestational age according to the Chinese Neonatal Network (1). Gestational age was determined by the mother’s last menstrual period or ultrasound measurement during early pregnancy and was confirmed by physical examination and ultrasonography when available. The flowchart of the study population is shown in **Figure 1**. All parents signed informed consent forms before participation. SGA infants born preterm (GA < 37 weeks) or postterm (GA > 42 weeks) and nonsingletons were excluded from the analysis. Besides, SGA infants with a dysmorphic features, congenital structural abnormalities of the organs, or chronic diseases had not been included. Additionally, individual measurements with unreasonable data were excluded in case of possible data-entry error. Only data from infants with anthropometric measurements for both weight and length at each of the follow-up age points were used in this analysis. Of these data, only those with at least one follow-up assessment during the period from 2–5 years were used. This study was approved by the Medical Ethics Committee of West China Second Hospital of Sichuan University.

### Measurements

Weight and length (height) were prospectively measured once a month in the first six months, once every 2 months from 6 months to 10 months, once every 3 months from 1 to 2 years, and once every six months from 2 to 6 years. Trained researchers measured physical indicators following strict protocols. Weight was measured using an electronic scale (Seca376; Seca Measuring System Co. Ltd., Hong Kong, China). Length for children under 3 years old was measured in the supine position using a measurement bed (Seca416; Seca Measuring System Co. Ltd.). Height for children older than 3 years was measured in a standing position by a measurement stadiometer. A dedicated person calibrated the measuring appliances regularly. Weight and length were separately assessed to the nearest 100 g and 0.1 cm, respectively. Body mass index (BMI) was calculated as weight in kg divided by the square of length in m.

### Growth trajectory

To group children with similar growth trajectory patterns according to their weight during the first 2 years of life, a latent class growth analysis (LCGA) was applied, which is a person-centered approach to classify individuals into distinct groups (25, 26). The 2006 World Health Organization (WHO) growth charts were used as a reference to calculate sex- and age-specific weight distributions prior to 2 years of age (27). Anthropometric indices of weight-for-age Z scores (WAZ) and height-for-age Z scores (HAZ) were calculated as indicators of the growth status of the children. The WAZ at each time point of 4 mo, 8 mo, 12 mo, and 24 mo was used for LCGA modeling. Participants were classified into 5 groups, WAZ < −1.28, − 1.28 ~ − 0.67, − 0.67~0.67, 0.67~1.28, and > 1.28, which corresponded to the 10th, 25th, 75th and 90th percentiles, respectively. The optimal number of growth trajectories (latent groups) was chosen according to the Bayesian information criterion. Lower Bayesian information criterion (BIC) values indicate a better fit. Moreover, the classification accuracy of the model was assessed with the entropy statistic. An entropy statistic >0.80 suggests sufficient accuracy of the model (range 0-1) (28).

Moreover, growth deviation was defined as being underweight or stunted, including children with Z score <−2 for weight or height, respectively, or being overweight or obese. BMI over the 85^th^ percentile and 95^th^ percentile was defined as overweight and obesity, respectively, in the current study. In addition, weight gain velocity between two target time points was indicated by different ΔWAZ degrees: i, ΔWAZ <−0.67 as crossing down one or more; ii, −0.67 ≤ ΔWAZ ≤0.67 as no crossing; iii, ΔWAZ > 0.67 to 1.28 as crossing up one; and iv, ΔWAZ >1.28 as crossing up two or more of the main weight percentiles of the WHO growth chart.

### Statistical analysis

We used latent class growth analysis (LCGA) implemented in Mplus software (version 8.0) to identify weight gain trajectories from birth to 2 years (28, 29). Statistical analysis was performed using SPSS (version 26.0). Cochran−Mantel−Haenszel χ^2^ was used to assess the differences in baseline characteristics among the 5 growth trajectory patterns. We used multiple logistic regression to examine the association between weight gain patterns and overweight/obesity adjusting for potential confounders, including sex, birthweight, gestational age, gravidity, parity, paternal height and BMI, feeding patterns between 0~4 mo. Furthermore, multiple logistic regression was applied to investigate the predictive impact of weight gain velocity from birth to 4 mo on overweight/obesity during 2-5 years with adjustment for the above confounding factors. Moreover, a receiver operating characteristic (ROC) curve was applied to test the sensitivity and specificity as well as the cutoff point for the predictive effect of weight gain in early life on later overweight/obesity. Statistical significance was defined as *P* < 0.05.

## Results

There were 296 term SGA infants with available length and weight data at birth, 4 mo, 8 mo, 1 y, 2 y and 2-5 y were finally included in the study. **Figure 1** shows the inclusion flow chart of the study population. Most demographic characteristics, including sex, gestation and birthweight, were not significantly different between SGA infants included and excluded from our study (**eTable 1**).

**Figure 2:**
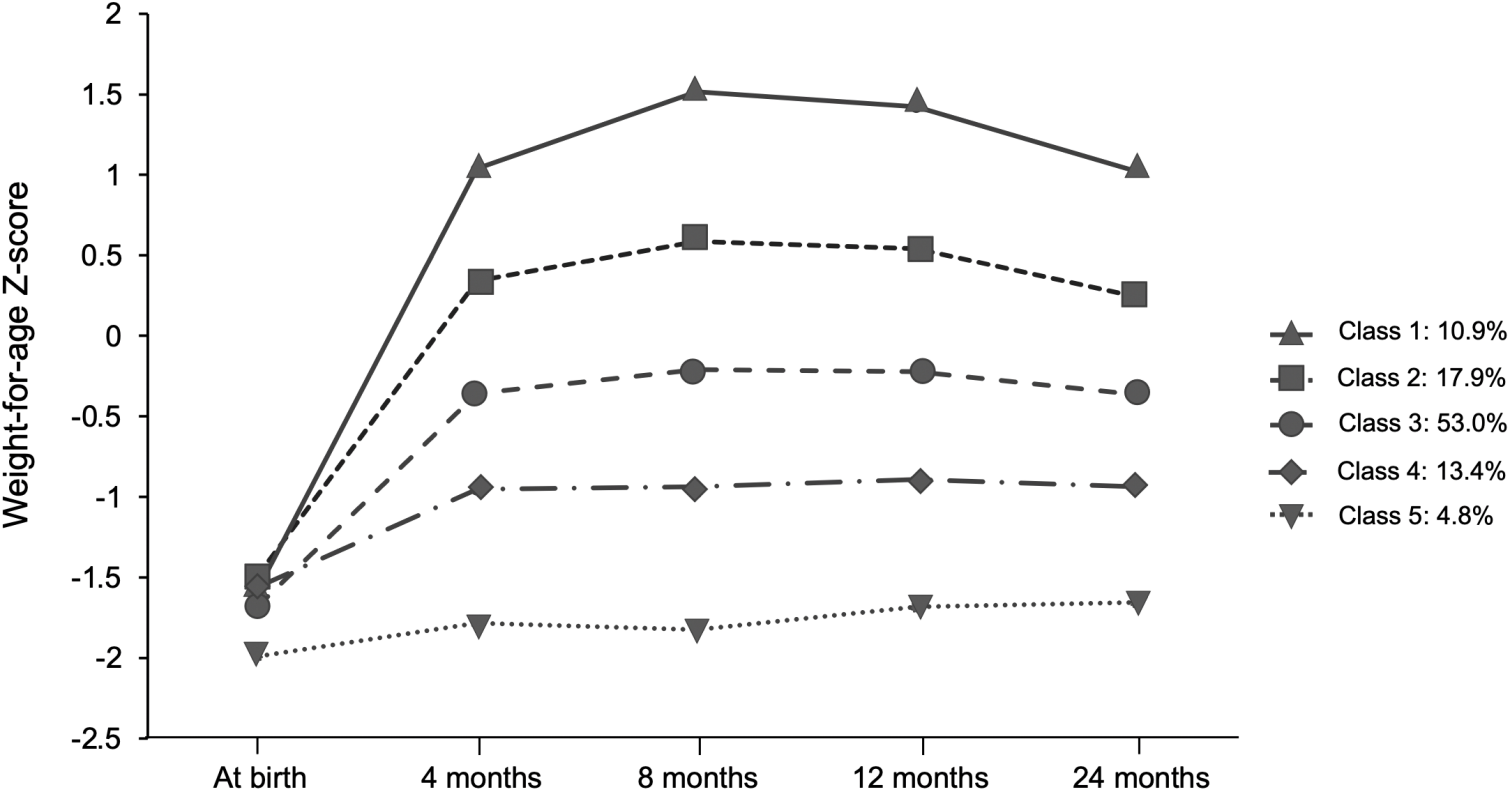
Trajectories of weight gain grouping classes in terms of SGA obtained by the latent class growth model (LCGA).

Based on the Bayesian information criterion in the LCGM, the BIC values were 4103.42, 3652.44, 3427.69, 3239.00, and 3293.68 when the population was divided into 2–6 categories, of which five were optimal grouping numbers for the minimum BIC. Five weight gain trajectories were identified, including excessive rapid catch-up growth (ERCG) (class 1, 10.9%), rapid catch-up growth (RCG) (class 2, 17.9%), appropriate catch-up growth (ACG) (class 3, 53.0%), slow catch-up growth (SCG) (class 4, 13.4%) and almost no catch growth (NCG) (class 5, 4.8%) (**Figure 2**). By comparing the baseline characteristics of SGAs in the 5 weight growth trajectories, we found that both birthweight and gestation played an important role (**Table 1**). SGAs with higher birthweight and late term were more prone to have weight gain trajectories from class 1 to 3 (*P*<0.01). Higher gravidity and parity were observed in the lower weight gain trajectories, but the difference was not significant (*P*>0.05). SGAs experienced class 1 and 2 weight gain trajectories were found having taller parents (*P*<0.01). Meanwhile SGAs with higher paternal BMI tend to have more rapid weight gain trajectories (*P*<0.01). Maternal BMI was highest in ERCG group, but with no significance (*P*>0.05). Additionally, feeding patterns between 0~4 mo was analyzed, and the proportion of formula feeding seemed to have a minor increase in class 1 and 2 weight gain trajectories. We further analyzed the disparities in ΔWAZ degrees during the first 4 mo of life among the five patterns. It was found that infants from classes 1 to 3 had a higher percentage of fast weight gain with ΔWAZ > 1.28 than the other two classes. Most infants in classes 4 and 5 had weight gain characterized by ΔWAZ from − 0.67 to 0.67 or ΔWAZ < − 0.67.

**Table 1:** The baseline characteristics of the SGA by weight gain class.

	Class 1 (ERCG)	Class 2 (RCG)	Class 3 (ACG)	Class 4 (SCG)	Class 5 (NCG)	*P* value
Number of children, n (%)	32 (10.8)	53 (17.9)	157 (53.0)	40 (13.5)	14 (4.7)	–
Male, n (%)	15 (46.9)	21 (39.6)	78 (49.7)	19 (47.5)	7 (50.0)	0.49
Birthweight, kg	2.55±0.24	2.54±0.23	2.51±0.24	2.45±0.22	2.31±0.26	<0.01
Birthweight Z score	-1.58±0.85	-1.49±0.49	-1.56±0.50	-1.68±0.47	-1.99±0.53	<0.01
Birthweight categories, n (%)						
BW<2.5kg	11 (34.3)	22 (41.5)	67 (42.7)	19 (47.5)	11 (78.6)	<0.01
2.5kg ≤ BW<3kg	21 (65.7)	31 (58.5)	90 (57.3)	21 (52.5)	3 (21.4)	
Gestation, wk.	39.00±1.21	39.02±1.28	38.88±1.21	38.64±1.15	38.28±1.13	<0.01
Gestation categories, n (%)						
Early term (37 wk. ≤ GA<40 wk.)	11 (34.4)	19 (35.8)	58 (36.9)	18 (45.0)	8 (57.1)	<0.01
Late term (40 wk. ≤ GA<42 wk.)	21 (65.6)	34 (64.2)	99 (63.1)	22 (55.0)	6 (42.9)	
Born type, n (%)						
Natural delivery	6 (18.8)	20 (37.7)	52 (33.1)	11 (27.5)	4 (28.6)	0.81
Cesarean	26 (81.2)	33 (62.3)	105 (66.9)	29 (72.5)	10 (71.4)	
Gravidity, n	1.20±0.63	1.30±0.82	1.41±0.81	1.30±0.63	1.47±1.10	0.34
Parity, n	1.00±0.00	1.04±0.19	1.07±0.26	1.09±0.29	1.14±0.48	0.13
Maternal height, centimeters	162.02±5.44	162.23±5.58	159.67±4.85	159.29±4.65	158.64±4.82	<0.01
Paternal height, centimeters	174.19±6.74	172.60±5.35	171.60±5.17	170.62±5.25	171.89±4.73	<0.05
Maternal BMI, kg/m^2^	21.19±3.02	20.77±2.93	21.10±2.55	20.82±3.42	20.75±2.84	0.88
Paternal BMI, kg/m^2^	23.55±2.34	24.30±3.27	23.83±2.57	22.80±3.58	22.81±2.75	<0.05
Feeding patterns between 0~4 mo						
Breast milk, n (%)	11 (35.6)	23 (43.4)	62 (39.7)	18 (45.4)	6 (39.3)	0.964
Mixed feeding, n (%)	15 (46.7)	21(39.6)	69 (44.1)	15 (37.8)	7 (46.4)	
Formula, n (%)	86 (17.8)	9 (17.0)	25 (16.2)	7 (16.8)	2 (14.3)	
ΔWAZ degrees between 4 mo. and birth, n (%)						
ΔWAZ <−0.67	0 (0.0)	0 (0.0)	0 (0.0)	1 (2.5)	2 (14.3)	–
−0.67 < ΔWAZ< 0.67	0 (0.0)	0 (0.0)	23 (14.7)	20 (50.0)	9 (64.3)	
0.67 < ΔWAZ<1.28	1 (3.1)	9 (17.0)	52 (33.1)	13 (32.5)	3 (21.4)	
ΔWAZ > 1.28	31 (96.9)	44 (83.0)	82 (52.2)	6 (15.0)	0 (0.0)	
Adverse growth outcomes, n (%)						
Overweight/ obesity	8 (25.0)	7 (13.2)	14 (8.9)	1 (2.5)	0 (0.0)	<0.01
Malnutrition	0 (0.0)	1 (1.9)	5 (3.2)	6 (15.0)	5 (35.7)	

ERCG, excessively rapid catch-up growth; RCG, rapid catch-up growth; ACG, appropriate catch-up growth; SCG, slow catch-up growth; NCG, almost no catch-up growth; BW, birthweight; ΔWAZ, ΔWeight-for-age Z-score degrees.

**Figure 3:**
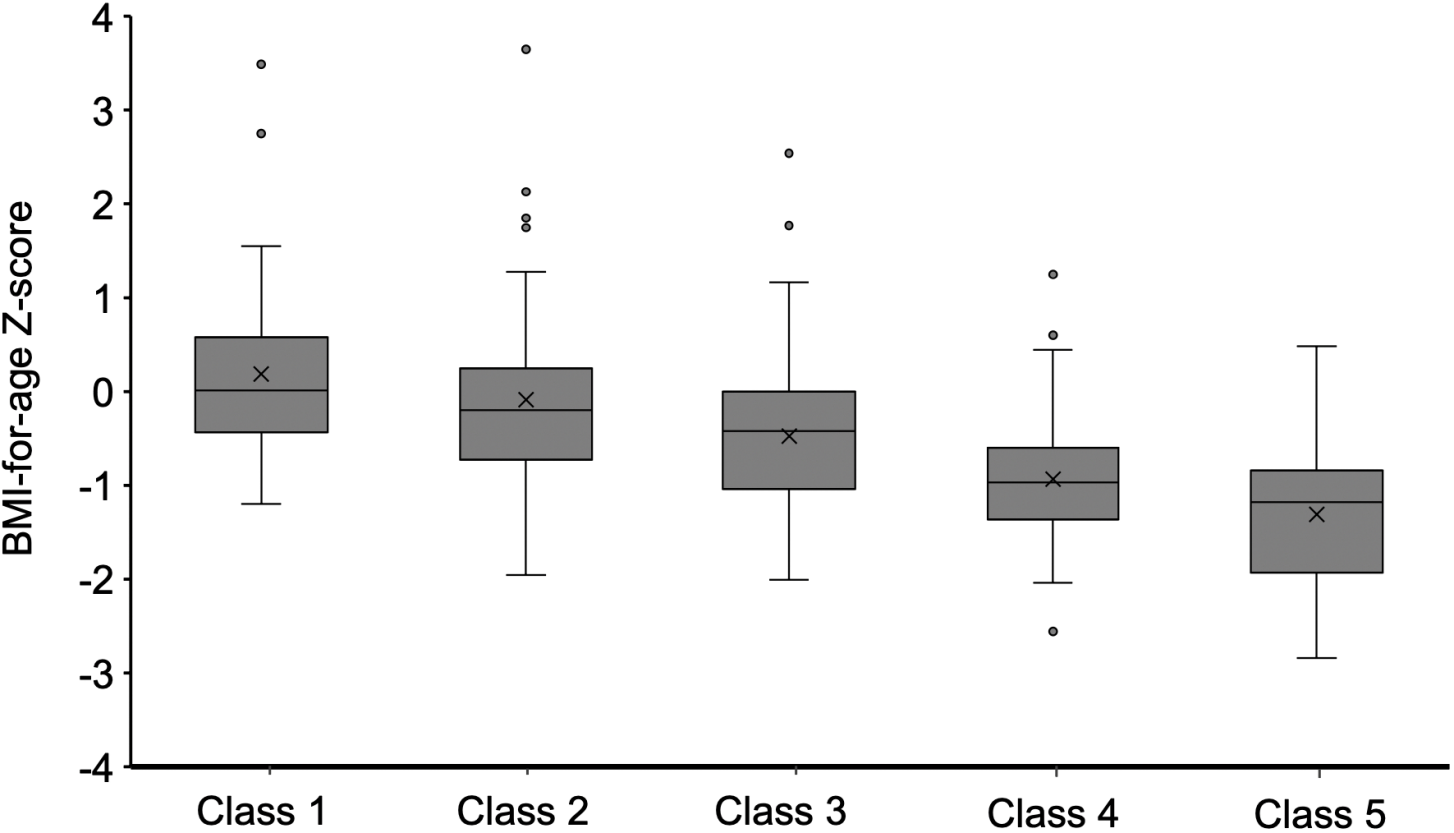
BMI Z score when participants aged 2–5 y were stratified by weight gain class in the first 2 years.

Furthermore, the study showed that weight gain classes in the first 2 years of life were associated with BMI for age z score and rate of overweight/obesity for SGA infants from 2-5 years (P<0.01). As shown in **Figure 3**, SGA infants in class 1 and class 2 had a higher BMI for age- and sex-specific Z score from 2–5 years. In addition, 25% of SGA infants in class 1 and 13.2% of SGA infants in class 2 were found to be overweight/obese at 2-5 years (**Table 1**). Nevertheless, 35.7% of SGA infants in class 5 were observed to be malnourished. After adjusting for confounding factors such as sex, birthweight, gestational age, gravidity and parity, paternal height and BMI, feeding patterns between 0~4 mo through multiple regression analysis, the results showed that the extremely rapid weight gain (class 1) of term SGA infants in the first 2 years of life increased the risk of overweight/obesity by 2.1 times at 2 to 5 years of age (aOR=2.1, 95% CI: 1.3~4.8; *P*<0.05). Besides, higher maternal height might be protective for childhood overweight/obesity at 2 to 5 years (aOR=0.823, 95% CI: 0.71~0.95; *P*<0.05).

**Figure 4:**
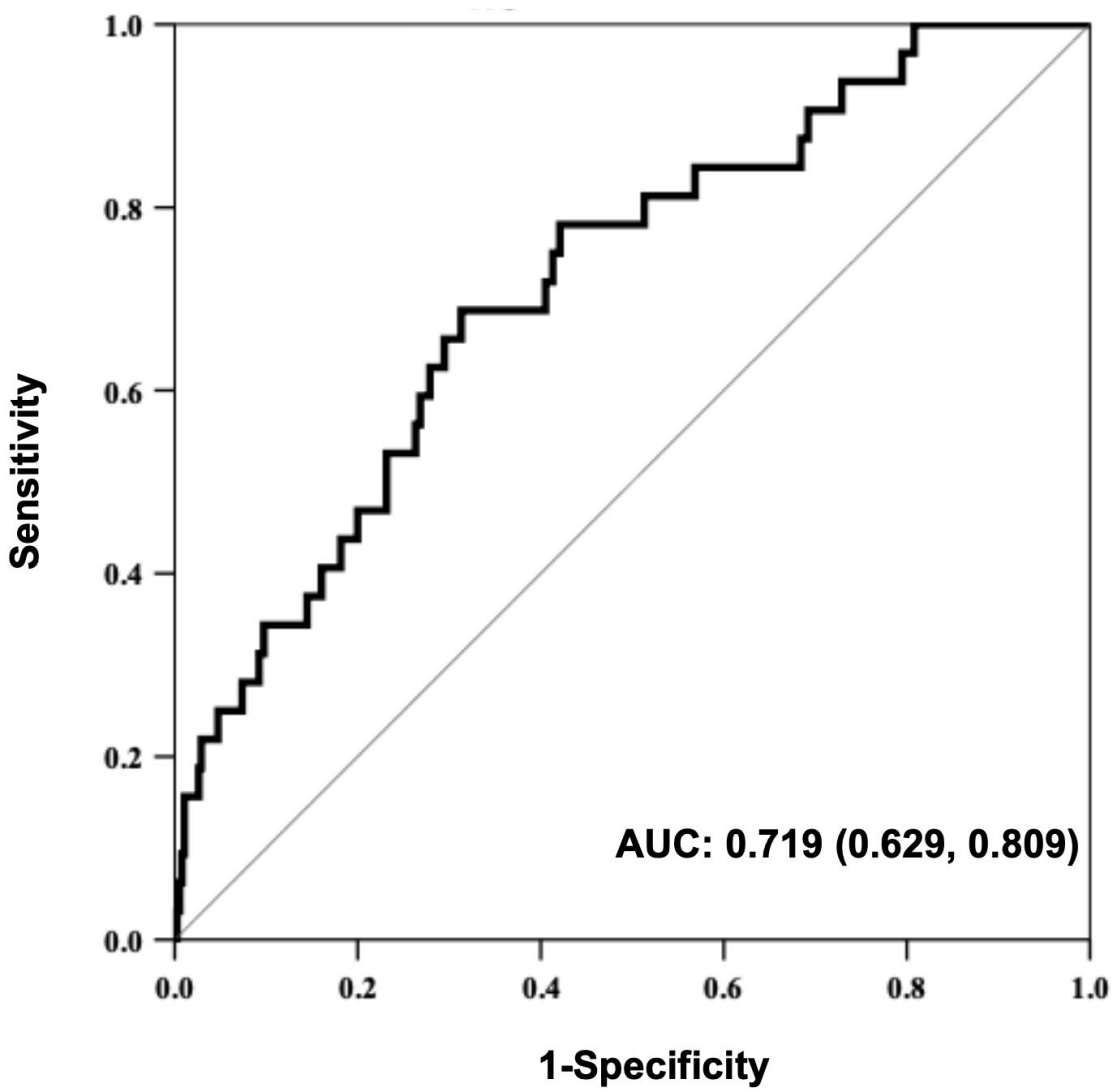
Receiver operating characteristic (ROC) curve for the predictive value of WAZ from birth to 4 months regarding overweight/obesity in SGA children aged 2~5 years.

We further explored the association of the ΔWAZ degrees of SGAs in early life with later overweight/obesity. When compared with the non-overweight/obese children, obviously higher ΔWAZ values from 0 to 4 mo (0.43 ± 0.12 vs. 0.26 ± 0.10, *P*<0.001) and 5 to 8 mo (0.07 ± 0.03 vs. 0.04 ± 0.02, *P*<0.01) were found in SGA infants with overweight/obesity in later life. After adjusting for confounding factors such as sex, birthweight, gestational age, gravidity and parity, paternal height and BMI, feeding patterns between 0~4 mo, the study showed that the risk of overweight/obesity between 2 and 5 years in term SGA infants was still related to the increment of ΔWAZ from 0 to 4 mo (aOR=3.2, 95% CI: 1.7~8.1; *P*<0.001). A receiver operating characteristic (ROC) curve was also established. The area under the curve (AUC) was 0.7, with a 95% CI from 0.6 to 0.8 (*P*<0.001) (**Figure 4**).

## Discussion

The best growth and nutritional strategy for term SGA is currently unclear and is likely to differ in different populations. To our knowledge, this is the first prospective birth cohort study to investigate the temporal relationship between early life excessive weight gain and childhood overweight/obesity in relatively low-middle income areas in West China. After adjustment for sex, birthweight, gestational age, gravidity, parity, paternal height and BMI, feeding patterns between 0~4 mo, the excessive rapid weight gain pattern from birth to 2 years was associated with an increased risk of childhood overweight and obesity at 2-5 years of life. Higher maternal height might be a protective factor. In addition, our findings suggest that the increment of ΔWAZ from 0 to 4 mo is a potential predictor of childhood overweight and obesity from 2-5 years of life. It is possible that pediatricians and parents should pay more attention to and ensure optimal early life weight gain, especially in the first 4 mo of life.

The weight gain pattern of term SGA infants is likely to differ in different populations and different income areas (30). This might lead to different nutrition strategies for term SGA infants. In the study, five weight gain patterns for term SGA infants from birth to 2 y were established in a low-middle income area in West China. The weight gain pattern was similar to that in a study performed in Shanghai, the most developed area in China (31). However, it revealed that the combined proportion of slow catch-up growth (SCG) and almost no catch growth (NCG) was much higher (18.2% vs. 13.6%), and the combined proportion of excessive rapid catch-up growth (ERCG) and rapid catch-up growth (RCG) was slightly lower (28.8% vs. 30.5%) than that among term SGA infants in high-income areas. This difference might be attributed to the relatively limited medical/health conditions and socioeconomic disparities in low-middle income areas. This finding suggests that different catch-up growth strategies should be adopted in different areas.

There are also many factors that affect catch-up growth, including socio-economic factors, genetic factors, maternal prenatal factors, maternal pregnancy complications, and postnatal feeding, diseases, etc (7, 32–34). First of all, the subjects of this study are basically from urban areas, and there is no significant difference between parents’ education levels. We further analyzed the effects of father’s height and BMI, mother’s height and prepregnancy BMI on SGA infants’ catch-up growth. The results showed SGA infants whose parents were taller or had higher BMI slightly tended to experience rapid weight catch-up growth. In the multivariate analysis, the height of mother was discovered a potential protective factor for obesity in full-term SGA infants aged 2-5 years. The genetic gene of higher height is helpful to promote SGA linear catch-up growth, which may be the potential reason (35, 36). In addition, this study found that the formula feeding within 4 months of term SGA infants with fast weight catch-up growth was slightly higher. The exclusive breastfeeding rate was lowest in ERCG group. One newly study reported that SGA preterm infants fed preterm formula had significantly larger negative change in weight and length z-scores between birth and discharge, when compared with fortified mother’s own milk (37). However, It was reported that breastfeeding may have positive effects on growth programming due to its nutrients’ energetic efficiency (38). The short-term and long-term benefits are mainly due to the adaptation of nutrient proportion, regulation of immunity, regulation of intestinal flora, etc (39–41). In this study, suspected congenital syndrome (with obvious special facial features and severe growth and development disorders), congenital organ abnormalities and chronic diseases that may seriously affect the growth and development of SGA infants were not included. This is helpful to reduce the influence of confounding factors on SGA infants catch-up growth pattern discrimination. After correcting important confounding factors, the excessive rapid weight gain pattern from birth to 2 years was still associated with an increased risk of childhood overweight and obesity at 2-5 years of life.

It has been illustrated by the concept of the Developmental Origins of Health and Disease (DOHaD) that children born with low birth weight or SGA have an increased risk for obesity, insulin resistance, and ultimately impaired glucose tolerance, type 2 diabetes, and cardiovascular disease later in life (14, 15, 42, 43). In this study, we found that the extremely rapid weight gain pattern (ΔWAZ>1.28) of term SGA infants from birth to 2 years was significantly related to the increased risk of overweight/obesity at 2-5 years in later life. The risk was approximately 2 times higher than that of term non-SGA infants. The pooled findings reviewed by Druet C et al. showed that each +1 unit increase in weight standard deviation (SD) scores between 0 and 1 year conferred a twofold higher risk of childhood obesity and a 23% higher risk of adult obesity, adjusted for sex, age and birthweight (44). A recent study from Denmark showed that rapid (ΔWAZ 0.67–1.34) and very rapid weight gain (ΔWAZ>1.34) among infants between 0 and 8–10 months of age dramatically increased the risk of overweight and obesity at 2 years (22–26 months) (45). The risks for overweight and obesity were nearly 3 and 7 times higher, respectively. Although there were some disparities in risks due to the different study designs and populations, it was clear that extremely rapid weight gain in early life indeed significantly increased the risk for overweight/obesity in later life. However, the trigger mechanisms remain unknown. The body fat content of a healthy full-term infant rises sharply from 10 to 14% at birth to 25 to 30% at 6 mo of age. Children born SGA had higher central adiposity regardless of their body size. Being SGA at birth could program excess abdominal fat deposition in children, which is a major component in the clustering of cardiovascular disease risk factors defining metabolic syndrome (MetS) (46). It is known that the early time is a critical period for growth and nutrition programming. The effect of excessive fat gain in the early stages on long-term nutrient metabolism deserves further investigation.

Dynamic changes in body weight have long been recognized as important indicators of risk for human health. Many population-based observational studies have shown that rapid weight gain during infancy, including a catch-up growth phenomenon or adiposity rebound in early childhood, predisposes a person to the development of obesity, type 2 diabetes, and cardiovascular diseases later in life (15). However, the exact timing of the rapid weight gain that contributes to these long-term risks continues to be debated. In this prospective study, we found that the fast increment of ΔWAZ from 0 to 4 mo was significantly related to the high risk of overweight/obesity at 2 and 5 years in term SGA infants. The risk was almost 3.2 times higher among these individuals. Additionally, the ROC curve suggested that ΔWAZ from 0 to 4 mo might be a potential predictor of the overweight/obesity risk of preschool children born at term and SGA. Similarly, a multicenter cohort study in the U.S. discovered that weight gain in the first 4 mo was associated with the OR of obesity at 7 years (47). Stettler N et al. found that African American infants gaining ≥1 WAZ unit in the first 4 mo were significantly more likely to be obese by age 20 (OR = 5.22) (48). In a Chinese cohort, an increase in WAZ in the first 3 mo was associated with BMI-Z at 7 years (49). Even increases in WAZ as early as the first 8 d of life have been associated with an increased risk of overweight and obesity in adulthood (50). The studies cited suggest that the first few weeks and months of life were particularly associated with later weight status (51). The disparity in research outcomes may result in part from ethnic differences, inconsistencies in research design, a lack of longitudinal and intervention studies, and appropriate unified indicators for catch-up growth. Previous studies have emphasized absolute weight gain, rarely addressing changes in body composition and failing to address fat distribution (subcutaneous vs. visceral). As also reviewed by Cho WK, the early fat increase in SGA infants may be the key factor affecting the occurrence of long-term cardiovascular metabolic diseases (46). Further prospective cohort multicenter studies evaluating fat growth should be designed. With regard to the mechanism(s) driving the correlation between excess weight gain in the first 4 mo and later obesity remain unknown. These months may be a critical time when metabolic programming can occur, similar to the *in utero* period, because infants’ organ systems still maintain considerable plasticity for adaptation to nutritional and environmental exposures. During the very early period, SGA infants are still malleable, and the gut is so permeable that milk/formulas can elicit significant endocrine responses. The combination of these factors potentially makes this time a vulnerable period (52).

### Limitations and prospects

This research has a number of limitations as well as strengths. First, the study failed to collect detailed information on detailed nutrition, which limited our analysis of the effects of nutritional factors on growth and development in SGA infants. Besides, the maternal obesity and weight gain during pregnancy were not well recorded. Second, we only included term SGA infants in the analysis. Thus, the results may not be applicable to preterm SGA infants or overdue SGA infants. Infants in these groups are thought to have different growth trajectories. Third, due to the limited duration of long-term follow-up, we analyzed whether the children were overweight or obese during the age of 2-5 years as the outcome. A better research design is needed in the future to ensure the integrity of long-term follow-up data. To identify an ideal growth trajectory, a longer follow-up period and more critical time points will be needed to assess the long-term impact of catch-up growth in childhood. These limitations aside, the strength of this research lies in the prospective study design with a relatively long follow-up time. Economic, geographical and ethnic factors are all potential factors affecting the growth and development of SGA infants. The risk of long-term chronic diseases among SGA infants requires early detection and timely intervention. However, to our knowledge, no such prospective cohort study has been conducted in Southwest China. This study has filled this gap to some extent. This study provides an important reference for the early health management of term SGAs in Southwest China.

## Conclusion

This study shows that excessive rapid catch-up weight growth in full-term SGA infants aged 0-2 years is an important risk factor for overweight/obesity at the age of 2-5 years. Scientific management of the weight increase in full-term SGA infants in early life, especially from 0-4 months, is the key to preventing the occurrence and development of overweight/obesity and its metabolic diseases in the later period. Children’s health care physicians should pay extra attention to this finding. Regular monitoring weight gain across the infant period represents a first step towards primary prevention of childhood obesity.

## Data availability statement

The original contributions presented in the study are included in the article/**Supplementary Material**. Further inquiries can be directed to the corresponding author.

## Ethics statement

The study protocol was approved by the medical ethics committee of West China Second University Hospital.

## Author contributions

Data extraction and curation: PL, SY. Statistical analysis: PL. Methodology: PL, FY. Validation: PL, DQ. Writing—original draft: PL. Writing—review and editing: all authors. All authors contributed to the article and approved the submitted version.

## Funding

This work was supported by the National Key Research and Development Program of China (No. 2019YFC0840702 to FY), the Science and Technology Bureau of Sichuan Province (No. 2020YFS0109 to Ping Li, No. 2021YFS0113 to YL), and the Clinical Discipline Development Fund of West China Second Hospital, Sichuan University (No. KL119 to PL).

## Acknowledgments

We appreciate the professional statistics staff of the School of Public Health for their guidance on the statistical methods. We thank all the medical staff involved in the follow-up.

## Conflict of interest

The authors declare that the research was conducted in the absence of any commercial or financial relationships that could be construed as a potential conflict of interest.

## Publisher’s note

All claims expressed in this article are solely those of the authors and do not necessarily represent those of their affiliated organizations, or those of the publisher, the editors and the reviewers. Any product that may be evaluated in this article, or claim that may be made by its manufacturer, is not guaranteed or endorsed by the publisher.
