# Supplementary material for: Early-life weight gain patterns of term small-for-gestational-age infants and the predictive ability for later childhood overweight/obesity: A prospective cohort study
Source: Front Endocrinol (Lausanne). 2022 Nov 22;13:1030216. doi: 10.3389/fendo.2022.1030216 (PMC9723138; doi:10.3389/fendo.2022.1030216)
Supplement: Supplementary file 1 [file Table_1.pdf]

eTable 1. Comparison of demographic characteristics of included and excluded term SGA in the study

	**Term SGA included**	**Term SGA excluded**	***P* value**
Number of children, n (%)	296 (27.8)	768 (72.2)	-
Male, n (%)	140 (47.3)	394 (51.3)	0.08
Birthweight, kg	2.47±0.23	2.51±0.26	0.14
Gestation, wk.	38.76±1.18	39.21±1.30	0.11
