# Supplementary material for: Early-life weight gain patterns of term small-for-gestational-age infants and the predictive ability for later childhood overweight/obesity: A prospective cohort study
Source: Front Endocrinol (Lausanne). 2022 Nov 22;13:1030216. doi: 10.3389/fendo.2022.1030216 (PMC9723138; doi:10.3389/fendo.2022.1030216)
Supplement: Supplementary file 2 [file Table_2.pdf]

eTable 2. The baseline characteristics of the SGA by weight gain class

	Class 1 (ERCG)	Class 2 (RCG)	Class 3 (ACG)	Class 4 (SCG)	Class 5 (NCG)	*P* value
Number of children, n (%)	32 (10.8)	53 (17.9)	157 (53.0)	40 (13.5)	14 (4.7)	-
Maternal educational level						
Junior high school and below, n (%)	1 (2.5)	2 (4.0)	6 (4.0)	2 (6.2)	1 (3.8)	-
High school, n (%)	0 (0.0)	2 (3.8)	8 (4.8)	3 (8.0)	1 (7.7)	
College or university degree, n (%)	26 (82.5)	40 (76.2)	114 (72.8)	27 (68.1)	9 (65.4)	
Master’s degree and above, n (%)	5 (15.0)	8 (16.0)	29 (18.4)	7 (17.7)	3 (23.1)	
Paternal educational level						
Junior high school and below, n (%)	1 (2.5)	2 (4.1)	4 (2.5)	2 (4.5)	1 (3.9)	-
High school, n (%)	0 (0.0)	2 (4.1)	9 (5.8)	3 (7.1)	1 (9.8)	
College or university degree, n (%)	26 (82.5)	40 (75.5)	110 (70.2)	29 (72.3)	11 (78.4)	
Master’s degree and above, n (%)	5 (15.0)	9 (16.3)	34 (21.5)	6 (16.1)	11 (7.8)	

Note:

Abbreviations: ERCG, excessively rapid catch-up growth; RCG, rapid catch-up growth; ACG, appropriate catch-up growth; SCG, slow catch-up growth; NCG, almost no catch-up growth.
